# Highlighting the resilience potential of marine protected areas in the face of coral bleaching with passive acoustic monitoring

**DOI:** 10.1098/rsos.241938

**Published:** 2025-07-09

**Authors:** Xavier Raick, Eric Parmentier, David Lecchini, Cédric Gervaise, Frédéric Bertucci, Guillaume Iwankow, Yannick Chancerelle, Gilles Siu, Lucia Di Iorio

**Affiliations:** ^1^ Laboratory of Functional and Evolutionary Morphology, FOCUS, University of Liège, Liège, Belgium; ^2^ K. Lisa Yang Center for Conservation Bioacoustics, Cornell University, Ithaca, NY, USA; ^3^ USR 3278 CRIOBE, PSL University, EPHE-UPVD-CNRS, Papetoai, French Polynesia; ^4^ Laboratoire d’Excellence ‘CORAIL’, Papetoai, French Polynesia; ^5^ Chorus Institute, Grenoble, France; ^6^ UMR MARBEC, University of Montpellier-CNRS-IFREMER-IRD, Sète, France; ^7^ GUMP Scientific Station, University of California Santa Barbara, Moorea, French Polynesia; ^8^ CEFREM, CNRS, UMR 5110, Université de Perpignan Via Domitia, Perpignan, France

**Keywords:** passive acoustic monitoring, coral reefs, French Polynesia, conservation bioacoustics, ecoacoustics, soundscape

## Abstract

Marine Protected Areas (MPAs) can increase the resilience of reef communities to disturbances, playing a role in sheltering biodiversity from climate-related impacts. To determine if the protection status allows for better resilience after coral bleaching events, we recorded soundscapes of eight reefs of Moorea Island (French Polynesia). We compared the biophony of MPAs to the one of adjacent non-protected zones recorded in 2015, before two bleaching events (2016 and 2019), to the one in 2021. Then, the biophony from 2021 was compared within and outside MPAs. We hypothesize that differences in the biophony between these periods vary within and outside MPAs. The main result is an increase in the nocturnal high frequency (2–22 kHz) mass phenomena of benthic invertebrates, observed at sites with higher coral cover post-bleaching compared to pre-bleaching: nocturnal power spectral density (PSD) and peak frequency of invertebrate sounds varied between 2015 and 2021. For fish sounds, no daytime difference was observed, while nocturnal PSD was higher in 2021. These observations reflect distinct bleaching histories. High-frequency PSD measurements and the associated frequency values demonstrated strong correlation with temporal changes in coral cover. We suggest including it in long-term reef monitoring due to its complementary nature with respect to classical methods.

## 1. Introduction

**Figure 1.**
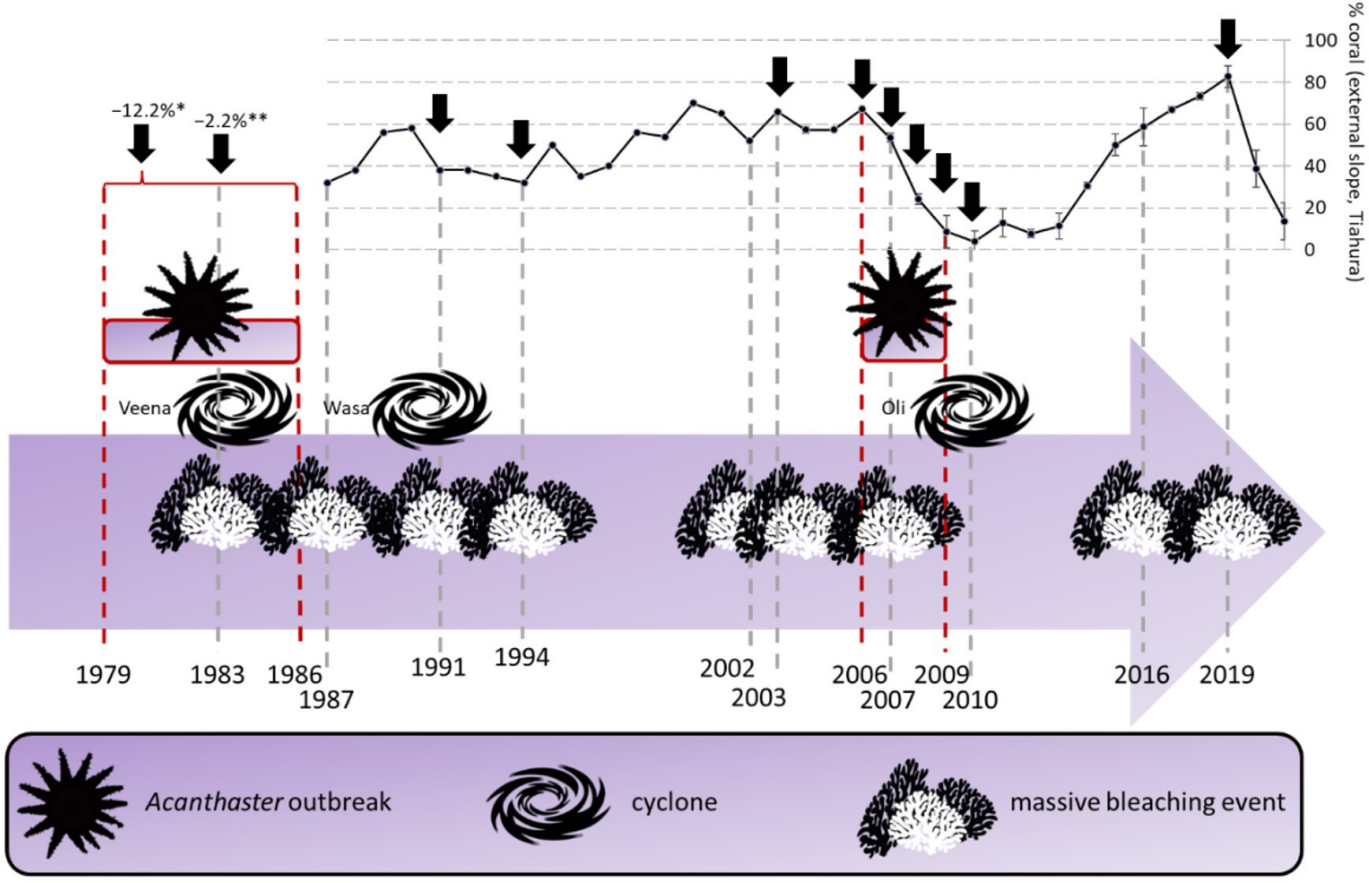
Timeline of negative events affecting the reefs of Moorea Island since 1979 and the percentage of corals at the external slope of Tiahura (Northwest) since 1987. Black arrows indicate decreases in coral cover related to the presented events (not visible for 1994 and 2010). The presence of a single event can cause a decrease in coral cover, but not always. References: 1979−1986 [15,16]; 1983 [2,17–19]; 1987 [2,18,19]; 1991 [1,19,20]; 1994 [20–22]; 2002 [20,22,23]; 2003 [23], Carroll *et al.* (unpublished data) in [14]; 2006−2009 [24–26]; 2007 [20,25,27]; 2010 [11,26]; 2016 [28–30]; 2019 [31]; coral cover between 1987 and 2003 [32]. *[15,16]; **[17]. Vertical bars since 2004 are standard deviation.

In this century, mass bleaching events caused by extended high water temperatures due to climate change are a major and increasing cause of degradation of coral reef ecosystems [1–10]. They alter a large variety of ecological processes within coral reefs (e.g. photosynthesis, reproduction, herbivory or predator–prey dynamics) [11] and can cause changes in the size of coral colonies, as well as the diversity and composition of the reefs’ associated fauna and flora [12]. In Moorea (French Polynesia), regular monitoring of coral cover has revealed that over the last four decades variation of the coral cover oscillated from <10% to >80% depending on the time and location [13,14] as a result of bleaching events occurring every 2−5 years [14] (figure 1). Between 2006 and 2010, a combination of bleaching events, crown-of-thorns sea star (*Acanthaster planci*) outbreaks and cyclones caused a loss of coral cover of around 60%, followed by a low recovery. In 2016, global warming combined with an El Niño event [33] affected coral reefs again [34], resulting in bleaching rates up to 44.4–61.2% [28]. However, the mortality was under 1% [28,29] and did not affect recruitment, and consequent coral cover increase [29]. In 2019, high sea temperatures provoked a new severe bleaching event [31] in Moorea Island. This time, bleaching was associated with a significant decrease in coral cover (figure 1).

To increase the resilience of coral reef communities to natural disturbances, including coral bleaching, coral diseases, *Acanthaster* outbreaks and cyclones [35], Marine Protected Areas (MPAs) have been promoted as effective management tools to protect biodiversity at local and global scales [13,36]. The *Plan de Gestion de l’Espace Maritime* (PGEM) is a network of eight MPAs around Moorea Island initiated in 2004 [37]. De Loma *et al.* [13] suggest that inside these MPAs, protection effects would be better detected in the outer slope habitat than in the barrier and fringing reefs, probably due to its less-variable geomorphology. Such results are rare, as ‘before’ data are not always available. Despite the existence of these rules regulating marine spaces and the collection of specific resources, there are few resources deployed to enforce sanctions in case of violations [37]. According to the literature, this is due to both the challenges in observing the infractions and the lack of penalties [37]. This raises questions about whether these MPAs can be considered effective.

While classic monitoring involves visual scuba-diving observations, passive acoustic monitoring (PAM) is emerging as a complementary method [38–40]. Recording sounds is well suited for non-invasive long-term ecosystem-based monitoring as it provides information about abiotic, biological and anthropogenic processes simultaneously [41–43]. Sounds emitted by animals can inform scientists on the presence, diversity and distribution of species, the phenology of biological events (e.g. diel, lunar and seasonal cycles of activity) and habitat quality [44–61]. Soundscapes of coastal reef ecosystems are dominated by passively emitted broadband transient sounds (BTS) from benthic invertebrates and fish sounds that form mass phenomena [62–64]. Mass phenomena are defined here as prolonged, continuous noises produced by the simultaneous sounds of many individuals (e.g. distant Pomacentridae sounds in shallow reefs) [65–68]. Sound pressure level (SPL) and acoustic indices generally applied to the coastal environment do not differentiate between different mass phenomena [69,70]. However, it has been shown that different habitats show different combinations of mass phenomena [59,71–73], and that these features can differ between species and behaviours [74,75]. Also, the level of these mass phenomena should be related to the abundance of sound-producing species.

In coral reefs, correlations have been found between the biophony (i.e. sounds emitted by animals within a particular ecosystem) and the coral cover [38]. The SPL of the coral reefs’ biophony has been shown to differ between MPAs and non-protected areas, with higher SPLs in MPAs, possibly reflecting the higher abundance of sound-producing species [38]. On the other hand, soundscape alteration has been shown to correlate with coral reef degradation state [76,77]. However, many of these studies focus on very extreme reefs (e.g. pristine reef versus highly degraded reef). It remains largely unknown if soundscapes reflect combined effects of protection measures and the impact of bleaching events. The objective of this study is to determine if PAM can be used to assess a resilience effect of MPAs to coral bleaching events. We hypothesize that differences in biophony will be observed before and after, both inside and outside MPAs. However, we also expect that the changes due to bleaching are less pronounced within MPAs due to their potential resilience effect.

## 2. Material and methods

The biophony of the external slope of the coral reefs of different sites in Moorea Island was recorded. First, data from 2021 were compared to data sampled in 2015 in MPAs and adjacent zones outside the MPAs. Then, MPAs and adjacent zones outside the MPAs recorded in 2021 were compared, enabling a more refined analysis of the differences between protected and non-protected areas. These two campaigns encompass two bleaching events (2016 and 2019) that caused an average coral cover loss of 10.2% [31].

### 2.1. Data collection

**Figure 2.**
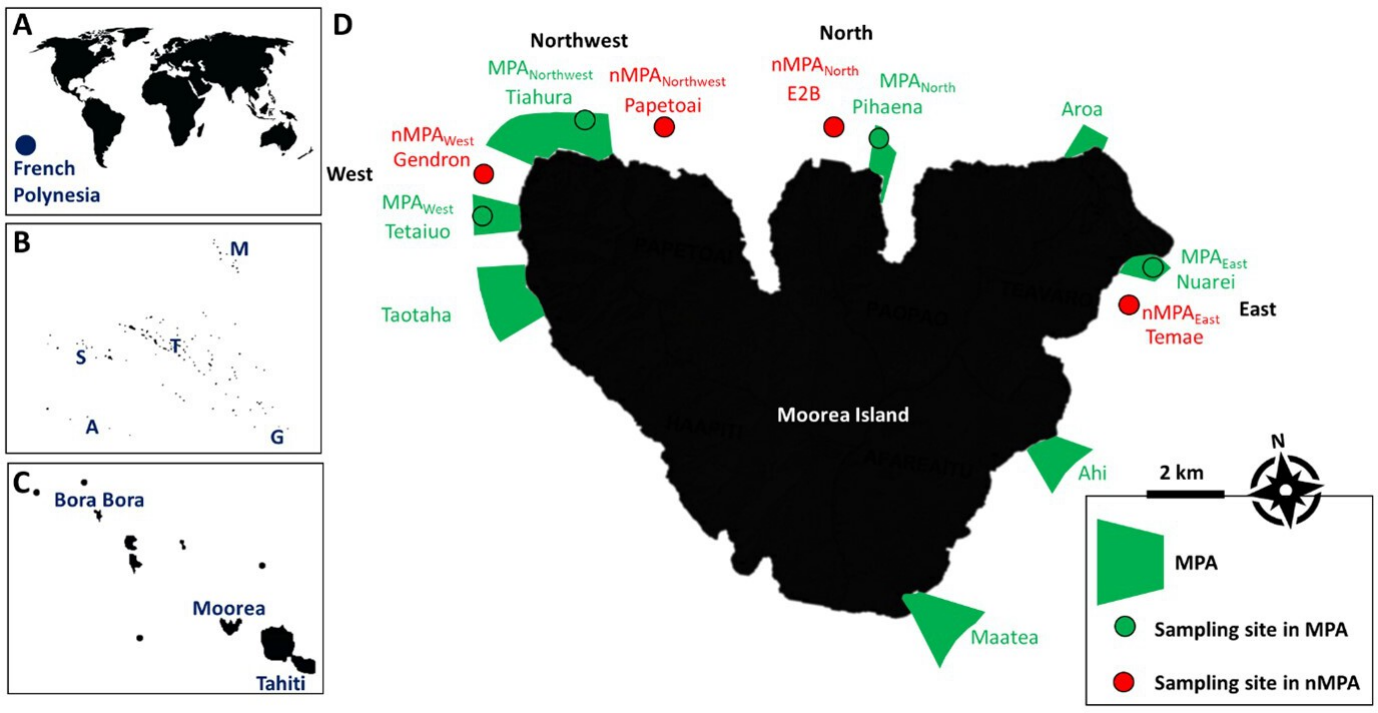
(A) Location of French Polynesia in the South Pacific Ocean. (B) French Polynesia consists of five archipelagos: Society (S), Marquesas (M), Tuamotu (T), Austral (A) and Gambier (G). (C) Zoom on the Society Archipelago. (D) Sampling sites around Moorea Island: two on the west coast, two on the northwest coast, two on the north coast and two on the east coast. MPA, marine protected area.

The study was conducted on the outer slope of the coral reefs of Moorea Island (Society Archipelago, French Polynesia). Two different periods were investigated: pre-bleaching events, i.e. before the bleaching event of 2016, and post-bleaching events, i.e. after the bleaching event of 2019. For both sampling periods, the same eight sites were surveyed at a depth of 10 m: four located within MPAs (Tetaiuo, Tiahura, Pihaena and Nuarei) and four outside MPAs (Gendron, Papetoai, Entre-deux-baies and Temae). MPAs and adjacent non-MPAs (nMPAs) were separated in West, Northwest, North and East couplets. To facilitate the reading of the article, the MPAs will be named MPA_West_, MPA_Northwest_, MPA_North_, MPA_East_, while the nMPAs will be named nMPA_West_, nMPA_Northwest_, nMPA_North_, nMPA_East_ throughout the document (figure 2).

To promote spillover of fish densities outside the MPAs and to integrate the several ‘associated municipalities’ (i.e. former municipalities that, after merging with others, lost their status as a territorial collectivity but retain some features, such as branch town halls), the decision was made to create small MPAs instead of a single large MPA [78]. Therefore, the largest width of these MPAs is 2 km. These MPAs are the only ones in French Polynesia for which an initial state has been described [78]. This island has the advantage of containing marine control areas, which are non-protected areas that are used as ‘controls’ in the monitoring of MPAs [79,80]. In all the MPA except MPA_East_, fishing is prohibited as well as any activity leading to degradation of the marine environment. In MPA_East_, fishing is allowed only with a line or for specific taxa (Gobiidae, Mullidae and some Scombridae). The MPA_Northwest_ includes a scientific reserve that is protected since 1993. Regulations within the MPAs include reduced boat speed, specific anchoring regulations, prohibitions on fishing, prohibition of shell collecting, protection of coral, prohibition of littering and discharging wastewater and prohibition of modification of the shoreline. These MPAs cover various marine habitats such as typical lagoons of high volcanic islands, fringing reef, reef channel and the external slope down to 70 m depth [78].

The initial sampling in 2015 was conducted over 2−3 days, including a full 24 h day that was used in this study. These data were not collected simultaneously [38]. An acoustic recorder (miniDSG, Loggerhead Instruments; Sarasota, FL, USA) connected to an HTI-96 hydrophone (sensitivity: −181 dB re 1 V µPa^−1^, gain: 10 dB) recorded sounds for 5 min h^−1^, of which 4.4 min were usable in this study, at a sampling rate of 48 kHz [38]. The second sampling took place over a period of 14 days in 2021. Acoustic recorders (SNAP, Loggerhead Instruments; Sarasota, FL, USA) connected to HTI-96 hydrophones (sensitivity between −170.1 and −169.6 dB re 1 V µPa^−1^; gain between 2 and 2.05 dB) continuously recorded sounds at a sampling rate of 44.1 kHz. Both data collections were performed between the end of January and the beginning of May, corresponding to the warm season (electronic supplementary material, table SP1). In 2021, four sites were sampled simultaneously (first set: MPA_North_, nMPA_North_, MPA_East_ and nMPA_East_; and second set: MPA_NorthWest_, nMPA_Northwest_, MPA_West_ and nMPA_West_).

### 2.2. Benthic cover and fish species

Benthic cover and fish diversity data were collected in February 2015 and in February 2021, as part of the scientific monitoring programme *Service National d’Observation CORAIL* (http://observatoire.criobe.pf). Data were available for all sites except for nMPA_Northwest_. Surveys were conducted 5 days before the full moon and always during daytime [81]. At each site, three transect lines (25 m long and 2 m wide, i.e. 50 m²) were laid parallel to the reef crest at a depth of 10 m (between 7 and 12 m in depth). Each transect line was surveyed twice: once for fish diversity and once for benthic cover. Three replicates were completed for each transect [78].

Fish were surveyed from the surface to the bottom. All observed fishes were identified to the species level. The entire transect line was surveyed to record the occurrence of very large or highly mobile fishes, while smaller individuals and more territorial species were counted in 5 m subsections. This process was repeated five times to cover the entire 25 m transect. This method ensured equal observation time for each portion of the transect [13].

The benthic cover was quantified every 50 cm using the *point intercept transect* method [82]. It was divided into 10 categories (sand, rubble, dead coral, pavement, *Asparagopsis* algae, *Halimeda* algae, *Turbinaria* algae, other macroalgae, *Millepora* fire coral and scleractinian corals; see electronic supplementary material, table SP2, for details) according to the procedure developed by Lison de Loma *et al.* [13]. Additionally, scleractinians were recorded to the genus level. The proportion (%) represented by each substrate type was calculated as the mean of the three transects. The total percentage of algae and the total percentage of coral cover were then calculated.

### 2.3. Acoustic analyses

For each site, data from 2021 were compared to the 24 h recording from 2015. To assess the mass phenomena produced by fish sounds, we focused on the low-frequency part of the soundscapes (below 2 kHz) because fish mainly emit sounds in this frequency range [64,83–86]. Separately, to assess the mass phenomena produced by benthic invertebrates, we focused on the high-frequency part of the soundscape (2–22 kHz) because this frequency range is known to contain BTS mainly produced by snapping shrimps [87–89]. In addition, soundscapes were divided into daytime (07.00 to 17.00) and nighttime (19.00 to 05.00). The protection effect (AMP versus nAMP) was first assessed using the data from 2021. These data were then compared to data from 2015. Each calendar day for each site was considered as a replicate.

For the analyses of mass phenomena of high-frequency benthic invertebrate sounds, the files from 2015 were subsampled from 48 to 44.1 kHz to match the sampling frequency of the files from 2021. To study low-frequency fish sounds, all the files were subsampled at 4 kHz. Then, sound pressure spectrum level graphs were generated with custom-made MatLab routines (MathWorks, Natick, MA, USA) (LFFT: 256 and 64, overlaps: 75% and 50% for low- and high-frequencies, respectively, windows: Kaiser). We decided to focus on the power spectral density (PSD) rather than on SPL because the SPL calculated between 20 Hz and 2 kHz, as sometimes found in the literature [38] when focusing on fish sounds, is affected by the low-frequency part of BTS and low-frequency mooring noise. PSD plots provide an overview of the levels of energy likely to occur at a given frequency and are source specific if the source is abundant [41]. The calculation of PSD on frequency bands that correspond to animals’ vocalizations is, therefore, linked to the abundance of the vocal activity of these animals.

**Figure 3.**
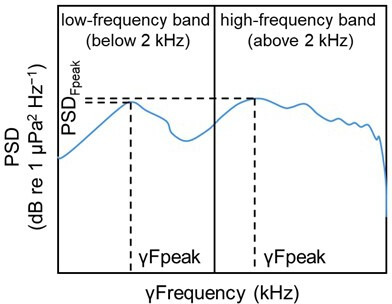
Schematic representation of the measurement of the highest power spectral density value (PSD_Fpeak_) and the frequency at which the power spectral density is maximal (γFpeak) on a median (Q0.50) PSD plot from a coral reef soundscape [64], divided into a low-frequency and a high-frequency band.

Two features were then measured on each median spectrum (figure 3): the highest PSD value (PSD_Fpeak_, in dB re 1 µPa^2^ Hz^−1^) and the frequency at which the PSD is maximal (γFpeak, in kHz) [90]. Based on the errors of the recording systems, when comparing data between the two systems, changes of less than 3 dB were considered as non-significant. Differences between averaging PSD_Fpeak_ data directly in dB re 1 µPa^2^ Hz^−1^ or converting them to linear values, computing the mean and then converting back to dB (∆) were well below the system’s error margin (low frequency and daytime: ∆ ≤ |0.03|; high frequency and nighttime: ∆ ≤ |0.11|; low frequency and nighttime: ∆ ≤ |0.44|; and high frequency and daytime: ∆ ≤ |0.12|).

PSD_Fpeak_ is a measure of the maximum intensity of sound energy within a given frequency range. This metric is critical because it provides insight into loudest part of the soundscape, which can have significant ecological effects. In fact, as sound level is linked to intraspecific communication and larval recruitment, measuring PSD_Fpeak_ helps identify if and how the acoustic environment is altered, which is crucial when assessing impacts such as bleaching. γFpeak indicates the frequency at which this peak intensity occurs. This is important because different species are sensitive to different frequency ranges. By identifying the frequency of the peak sound energy, we can determine how changes in the soundscape might affect species that rely on specific frequencies for communication or recruitment. These metrics are particularly suitable for this study because they provide a quantitative measure of both sound intensity (PSD_Fpeak_) and its frequency characteristics (γFpeak). This dual approach enables a comprehensive assessment of how sound energy distribution changes in response to environmental conditions. Other metrics, such as average SPL across broad frequency ranges or temporal changes in sound, might not capture these specific and critical aspects of the acoustic environment as effectively. Therefore, PSD_Fpeak_ and γFpeak are chosen for their ability to directly relate to ecological impacts and their sensitivity to changes in the soundscape that are relevant for understanding the effects of bleaching and MPAs.

### 2.4. Statistical analyses

#### 2.4.1. Pre-bleaching versus post-bleaching in the low-frequency band

Analyses of covariance (ANCOVAs) were carried out for each response variable (diurnal PSD_Fpeak_, nocturnal PSD_Fpeak_, diurnal γFpeak and nocturnal γFpeak). The complete dataset (2015 (*n* = 8) and 2021 (*n* = 112)) was used to assess the interaction effect of year and protection (year (two levels: 2015 or 2021) × protection (two levels: MPA or nMPA), random = location (West, North, Northwest and East) and moon phase = covariate). Each calendar day for each site was considered as a temporal replicate. Only differences greater than |3| dB, i.e. the internal measurement error, were considered significant. Violin plots were used for visualization (package ggplot2, function geom-violin). A canonical correspondence analysis (CCA) on all the data was conducted to test the influence of benthic cover features (percentage of coral and percentage of algae) and acoustic features (diurnal PSD_Fpeak_, nocturnal PSD_Fpeak_, diurnal γFpeak and nocturnal γFpeak) on fish community composition (package vegan, function cca). Protection (MPA or nMPA) and the year (2015 or 2021) were added as grouping variables to the ordination plot to visualize their relationships to fish community and features (function ordiellipse, with a 95% confidence interval).

#### 2.4.2. Pre-bleaching versus post-bleaching in the high-frequency band

The same ANCOVAs and violin plots that were used for the low-frequency band were applied to the high-frequency band. A CCA was performed to test the influence of benthic cover features (percentage of coral, algae, pavement, rubble or sand) and acoustic features (diurnal PSD_Fpeak_, nocturnal PSD_Fpeak_, diurnal γFpeak and nocturnal γFpeak) on coral cover composition (package vegan, function cca). The protection variable (MPA or nMPA) and the year (2015 or 2021) were added to the ordination plot to visualize their relationships with coral genera and features (function ordiellipse, with a 95% confidence interval).

#### 2.4.3. MPAs versus nMPAs in the low-frequency band

All statistics were performed using R software version 4.1.1 (R Core Team, 2021) with the significance level set at *α* = 0.05. Acoustic data were averaged to obtain one datum per site per night or per daytime period. ANCOVAs (package nlme, function lme) followed by type II tests (function Anova) were conducted for each response variable (diurnal PSD_Fpeak_, nocturnal PSD_Fpeak_, diurnal γFpeak and nocturnal γFpeak) on the data collected in 2021 to estimate the presence or absence of protection as main effect (protection (two levels: MPA or nMPA)), site as random effect (location (West, Nortwest, North and East)) with moon phase as covariate. Analyses of variance were used to compare fish abundance, fish species richness (number of different species present) and percentage of cover features between MPAs and nMPAs with location as a random factor. Violin plots were used for visualization (package ggplot2, function geom-violin). A CCA was conducted to investigate the influence of benthic cover features (percentage of coral and percentage of algae) and acoustic features (diurnal PSD_Fpeak_, nocturnal PSD_Fpeak_, diurnal γFpeak and nocturnal γFpeak) on fish assemblages (package vegan, function cca). The CCA was used to understand the link between fish species and to relate these species to combinations of the features [91].

#### 2.4.4. MPAs versus nMPAs in the high-frequency band

The same ANCOVAs and violin plots that were used for the low-frequency band were performed for the high-frequency band. A CCA was conducted to investigate the influence of benthic cover features (percentage of coral and percentage of algae) and acoustic features (diurnal PSD_Fpeak_, nocturnal PSD_Fpeak_, diurnal γFpeak and nocturnal γFpeak) on coral cover composition (package vegan, function cca).

## 3. Results

### 3.1. Pre-bleaching versus post-bleaching in the low-frequency band

**Figure 4.**
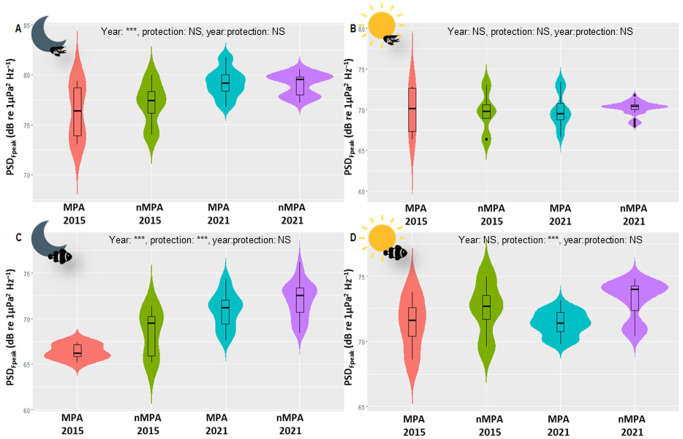
Violin plots of PSD_Fpeak_ in MPAs and nMPAs in 2015 and 2021. (A) Nocturnal high-frequency band, (B) diurnal high-frequency band, (C) nocturnal low-frequency band and (D) diurnal low-frequency band. The medians are represented by the thick horizontal lines in the box plots. Each whisker extends up to 1.5 × IQR, where IQR is the interquartile range, i.e. the distance between the first and third quartiles. The violins help visualize the distribution of the data. Note the difference in the vertical scales. Sun icons indicate daytime, moon icons indicate nighttime, fish icons represent fish sounds and shrimp icons represent sounds from benthic invertebrates.

**Figure 5.**
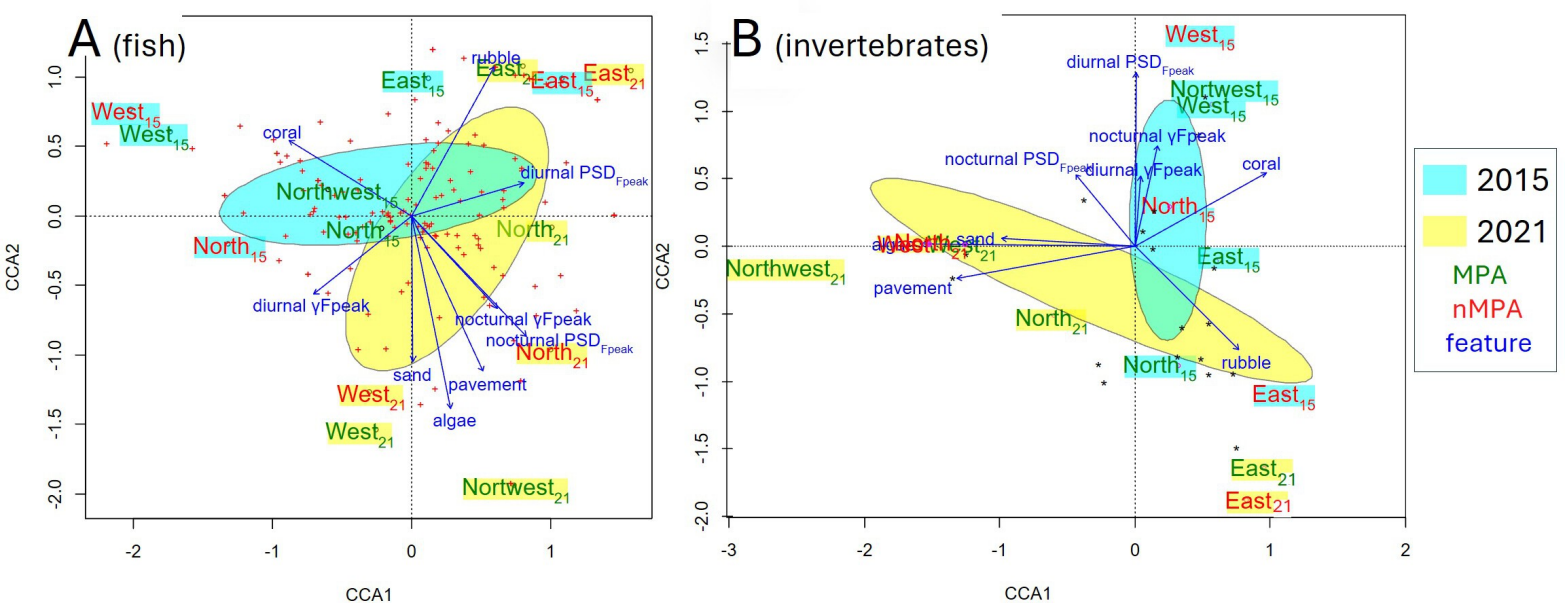
Canonical correspondence analysis ordination plot based on Bray–Curtis dissimilarities of relative abundances of (A) fish species and (B) coral/algae genera between 2015 (cyan) and 2021 (yellow). Crosses show (A) fish species and (B) coral/algae genera. Blue arrows show the influence of benthic cover and (A) acoustic low-frequency features and (B) high-frequency features. Sites are indicated in green (MPAs) and red (nMPAs). Ellipses are 95% interval: in cyan (2015) and yellow (2021).

In the low-frequency band, only nocturnal PSD_Fpeak_ varied between 2015 and 2021 (*χ*^2^ = 44.82, d.f. = 1, *n* = 120, *p* < 0.0001), with an average increase of 4.07 dB re 1 µPa^2^ Hz^−1^ in 2021 (electronic supplementary material, tables SP5 and SP6). γFpeak and daytime PSD_Fpeak_ variations between the two years were not significant (electronic supplementary material, table SP6). An acoustic mass phenomenon with a higher PSD may indicate increased acoustic activity of fish during the period sampled in 2021 compared to 2015. This increase was observed at all sites (between 2.56 and 6.44 dB re 1 µPa^2^ Hz^−1^) with maximal values at MPA_Northwest_. This increase was higher in MPAs than in nMPAs (4.55 versus 3.58 dB re 1 µPa^2^ Hz^−1^, figure 4). Diurnal PSD_Fpeak_ did not statistically differ between 2015 and 2021 when considering all the sites together. A significant difference (i.e. greater than 3 dB) was observed only for MPA_Northwest_ (3.83 dB re 1 µPa^2^ Hz^−1^). Between 2015 and 2021, the CCA highlighted smaller changes for eastern sites than for all the other sites (figure 5A). These displacements are related to a decrease in coral cover and an increase in algae and pavement percentage. All the sites, except eastern sites, had a reduction in coral cover (between −34% and −3%). Eastern sites were the only two with an increase in coral cover (between +10% and +21%). All the sites had an increase in algae cover. All the sites, except the eastern sites, had an increase in pavement cover.

### 3.2. Pre-bleaching versus post-bleaching in the high-frequency band

**Figure 6.**
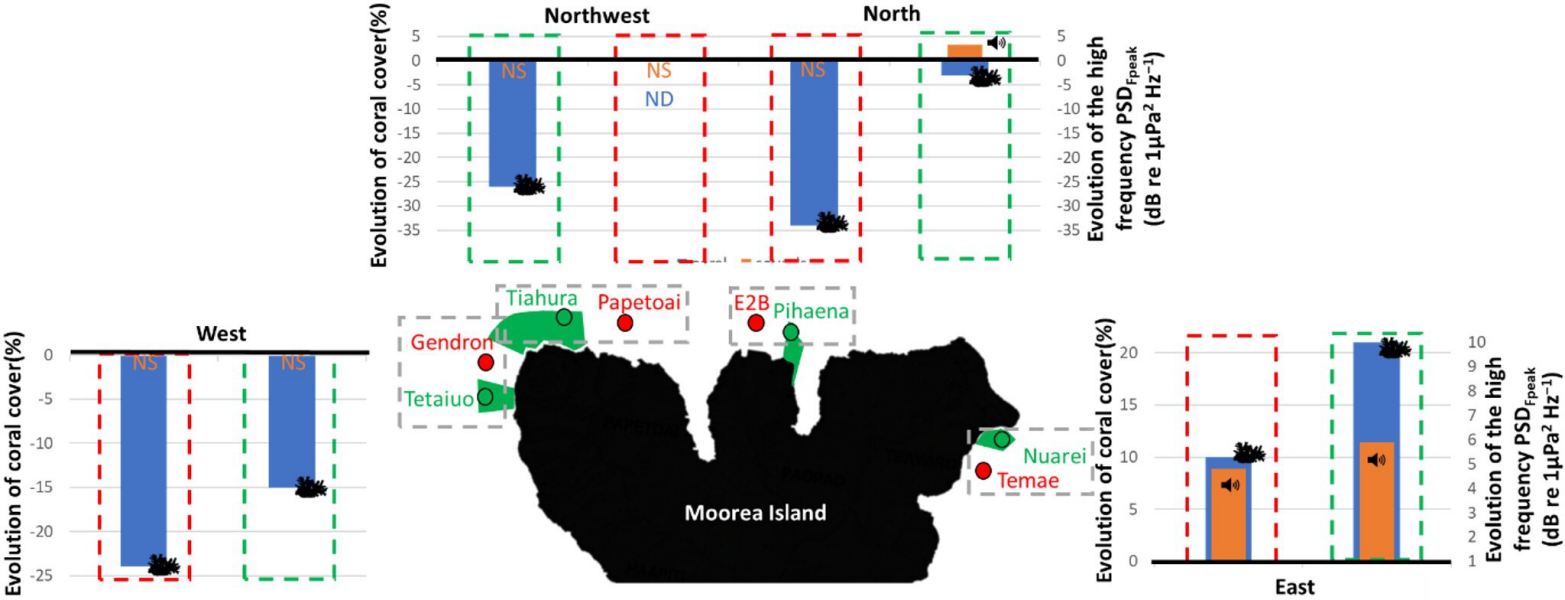
Illustration of loss/gain in coral cover and high-frequency PSD_Feak_ (respectively in blue and orange) between 2015 and 2021. NS, non-significant; ND, no data. The loss/gain in coral cover was calculated by subtracting the percentage of coral cover in 2015 from that in 2021.

In the high-frequency band, nocturnal PSD_Fpeak_ and γFpeak varied between 2015 and 2021 (*χ*^2^ = 23.35 and 5.62, d.f. = 1, *n* = 120, *p* < 0.0001 and *p* = 0.018, respectively; figure 5B). There was an interaction between protection status and the year for both diurnal and nocturnal γFpeak (*χ*^2^ = 32.64 and 14.79, d.f. = 1, *n* = 120, *p* < 0.0001 and 0.00012). No changes of PSD_Fpeak_ above the |3| dB error margin of the system were observed (increase of 2.88 in MPAs versus 1.96 dB re 1 µPa^2^ Hz^−1^ in nMPAs). Nocturnal PSD_Fpeak_ variations > |3| dB were only observed on the three eastern sites (increase of 3.41, 4.79 and 5.93 dB re 1 µPa^2^ Hz^−1^; figure 6). Diurnal PSD_Fpeak_ variations > |3| dB were only observed at MPA_Northwest_, with a decrease of 3.13 dB re 1 µPa^2^ Hz^−1^. The examination of the CCA ordination plot reflects this decrease, also associated with a loss in coral cover by a factor of 3 and an increase in algae by a factor of 6. Diurnal PSD_Fpeak_ seems to be more associated with coral cover than nocturnal PSD_Fpeak_.

### 3.3. MPAs versus nMPAs in the low-frequency band

**Figure 7.**
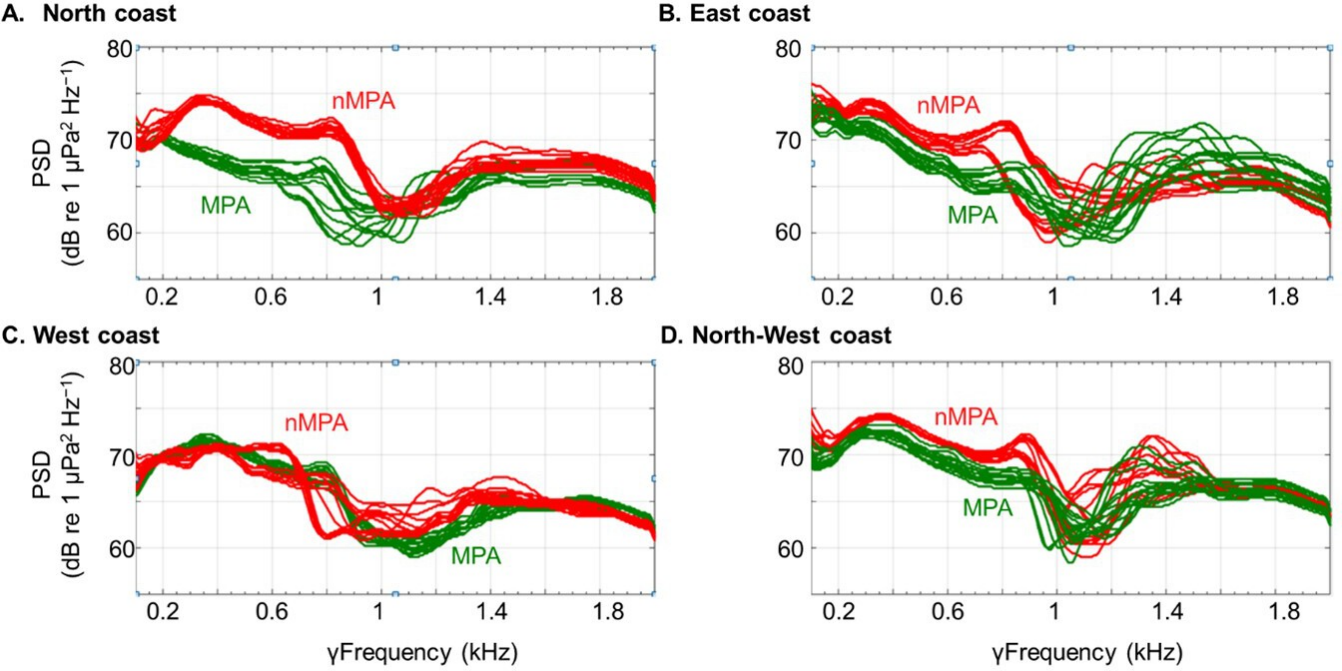
PSDs in the low-frequency band (<2 kHz) during daytime in 2021. For each coast (North, East, West and Northwest), MPAs are in green and nMPAs areas are in red. For each condition, 14 temporal replicates are represented.

**Figure 8.**
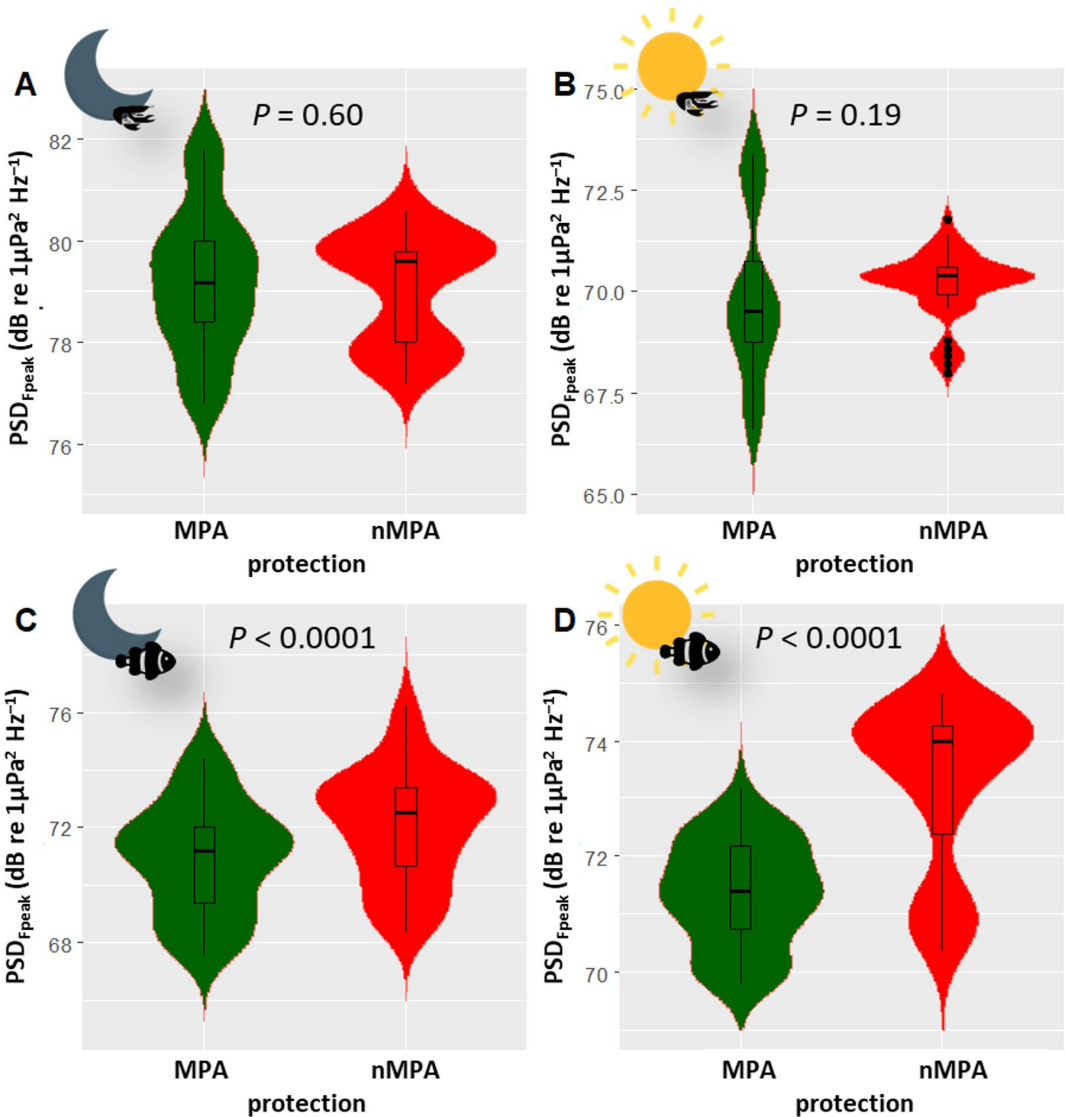
Violin plots of PSD_Fpeak_ in MPAs and nMPAs in 2021. (A) Nocturnal high-frequency band, (B) diurnal high-frequency band, (C) nocturnal low-frequency band and (D) diurnal low-frequency band. The medians are represented by the thick horizontal lines in the box plots. Each whisker extends up to 1.5 × IQR, where IQR is the interquartile range, i.e. the distance between the first and third quartiles. The violins help visualize the distribution of the data. Note the difference in the vertical dB scale. Sun icons indicate daytime, moon icons indicate nighttime, fish icons represent fish sounds and shrimp icons represent sounds from benthic invertebrates.

**Figure 9.**
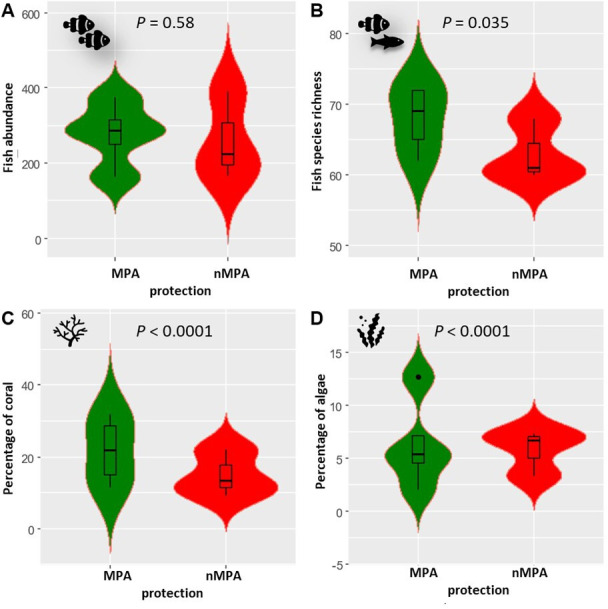
Violin plots of ecological features in 2021. (A) Fish abundance, (B) fish species richness, (C) % of coral cover and (D) % of algae cover. The medians are represented by the thick horizontal lines in the box plots. Each whisker extends up to 1.5 × IQR, where IQR is the interquartile range, i.e. the distance between the first and third quartiles. The violins help visualize the distribution of the data. Note the difference in the vertical scales.

**Figure 10.**
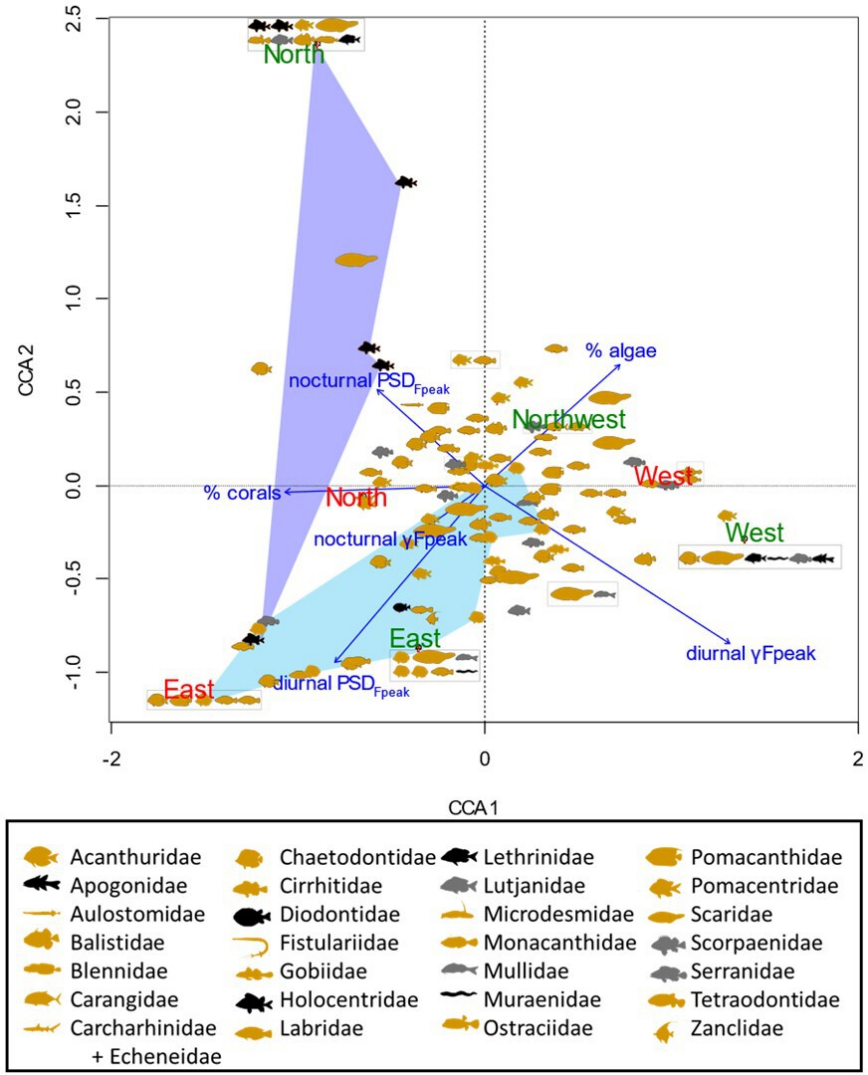
Canonical correspondence analysis ordination plot of fish assemblages based on Bray–Curtis dissimilarities of relative abundances of fish species. Acoustic features were calculated in the low-frequency band (<2 kHz). Blue arrows show the influence of benthic cover and acoustic low-frequency features. Shapes were used per fish family (one datapoint per species). In yellow: diurnal families; in grey: diurnal and nocturnal families; in black: nocturnal families. Grey boxes gather species with the same CCA coordinates. Sites are indicated in green (MPAs) and red (nMPAs).

In the low-frequency band (below 2 kHz) in 2021, diurnal γFpeak was lower in MPAs than in nMPAs (292 versus 355.5 Hz, *χ*^2^ = 111.62, d.f. = 1, *p* < 0.0001; electronic supplementary material, table SP3; figure 7). PSD_Fpeak_ was lower in MPAs than in nMPAs, during both the day and night (*χ*^2^ = 110.51 and 19, d.f. = 1, both *p* < 0.0001; figure 8C,D). Fish abundance was equivalent between MPAs and nMPAs (*χ*^2^ = 0.31, d.f. = 1, *p* = 0.58; figure 9; electronic supplementary material, table SP4), but MPAs showed a higher fish species richness (*χ*^2^ = 4.45, d.f. = 1, *p* = 0.035) and a higher percentage of coral and algae cover (*χ*^2^ = 21.86 and 48.47, d.f. = 1, both *p* < 0.0001) than nMPAs. The CCA ordination plot reveals that diurnal PSD_Fpeak_ displayed an opposite trend compared to the percentage of algae, implying that fish mass phenomena were louder in sites with a low algae cover (figure 10). Some taxa like Chaetodontidae (figure 10, cyan polygon) were clearly reef associated. The majority of Holocentridae species (83.3%) were restricted to the upper left corner of the plot (figure 10, dark blue polygon). This area coincides with intense nocturnal PSD_Fpeak_ values. These results coincide with a known high nocturnal feeding activity of Holocentridae [92]. Positive CCA1 values are associated with the west coast while negative CCA1 values are associated with the east coast.

### 3.4. MPAs versus nMPAs in the high-frequency band

**Figure 11.**
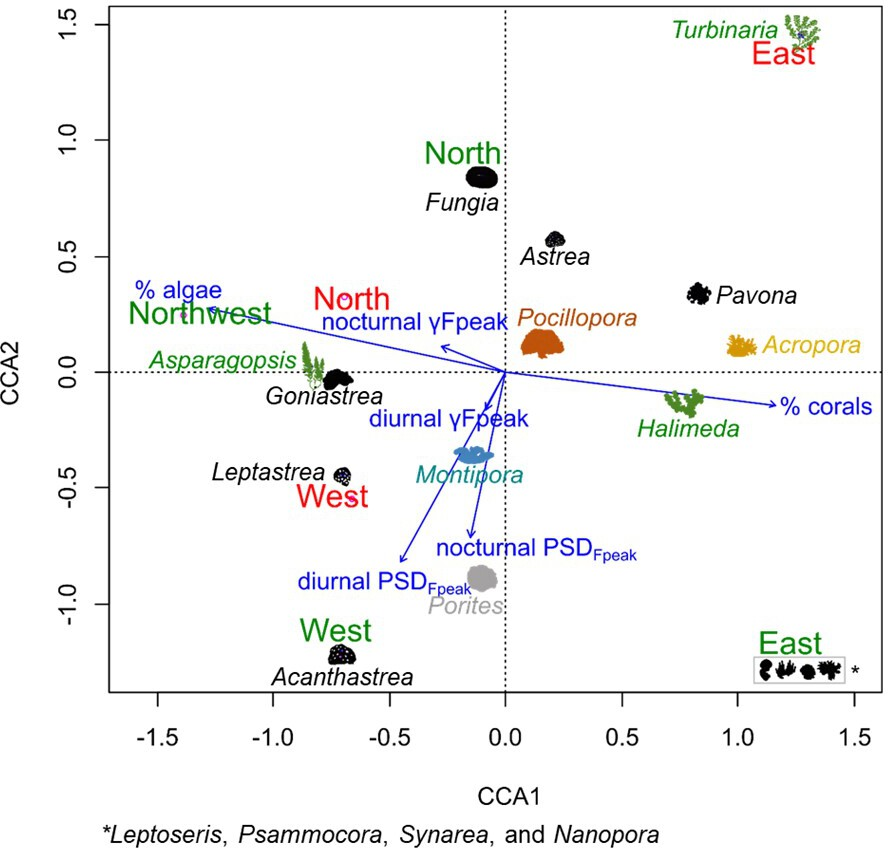
Canonical correspondence analysis ordination plot of the coral/algae assemblages based on Bray–Curtis dissimilarities of relative abundances of coral/algae genera. Acoustic features were calculated in the high-frequency band (2–22 kHz). Shapes were used per coral/algae genus. Blue arrows show the influence of benthic cover and acoustic high-frequency features. Sites are indicated in green (MPAs) and red (nMPAs).

In the high-frequency band (2–22 kHz) in 2021, γFpeak was lower in MPAs than in nMPAs during both the day and the night (daytime: 5.55 versus 4.96 kHz, nighttime: 5.28 versus 4.60 kHz, *χ*^2^ = 145.74 and 103.84, both *p* < 0.0001; figure 8). This difference is still within the known range of snapping shrimps’ sound emissions. PSD_Fpeak_ values were not statistically different between MPAs and nMPAs (*χ*^2^ = 0.27 and 1.69, *p* = 0.60 and 0.19). Positive CCA1 values were associated with the total percentage of corals and the presence of *Acropora*, a genus of usually arborescent/caespitose or tabular corals (figure 11). The percentage of coral cover was higher on eastern sites (figure 11). The total percentage of corals did not explain PSD_Fpeak_ which would have been expected if the presence of snapping shrimps had been the same irrespective of coral cover composition. Negative values of CCA2 were associated with PSD_Fpeak_ and the presence of *Porites*, a genus of stony corals, known to host snapping shrimps [93].

## 4. Discussion

This study finds that the differences in the high-frequency band of soundscapes are the illustrations of the observed protection effect that MPAs confer to coral cover after bleaching events. This is of crucial importance as degraded reef sounds are less attractive during the recruitment process, resulting in a reduced settlement, posing a threat to the recovery of degraded reefs [94,95]. In addition, it is now clear that soundscapes can be degraded when severe damages occur on the reef [94]. In Polynesia, after the bleaching events, an increase in nocturnal high-frequency mass phenomena of benthic invertebrates was observed only at sites with higher coral cover. This relationship could be explained by the dominance of snapping shrimp sounds in this frequency band during nighttime. This confirms the positive relationship reported between snapping shrimp sounds and coral cover [76,77]. In addition, the high-frequency spectral component also reflected the geographical bleaching history of the island with the west coast being more affected than the east coast (cf. §3.1). Therefore, PAM may be used not only for monitoring the correlation between coral cover and biophony but also for tracking long-term changes and assessing the evolution of coral cover following events such as bleaching.

### 4.1. Pre-bleaching versus post-bleaching

When comparing pre-bleaching and post-bleaching soundscapes, a significant increase in the nocturnal high-frequency biophony was found on the eastern sites. These findings are in agreement with visual observations showing an east–west gradient in coral cover, with eastern sites being the only ones with a higher coral cover in the post-bleaching period compared to the pre-bleaching period [28–31]. In 2016, bleaching was more severe on the west coast (up to 100% *Acropora* colonies bleached) and on the north coast (twice the number of *Pocillopora* colonies bleached compared to the other coasts) [30]. The situation was more mitigated in the north coast than in the west coast [28,29]. In 2019, the bleaching event was shorter on the east coast compared to the west and north coasts [31]. Consequently, this resulted in a higher coral mortality on the north coast, intermediate mortality on the west coast and lower mortality on the east coast. During the day, a significant decrease in PSD_Fpeak_ was observed at MPA_Northwest_. This is consistent with the high coral mortality observed in this area (Service National d’Observation CORAIL). This positive relationship between high PSD_Fpeak_ values and coral cover agrees with the SPL observations made by Bertucci *et al.* [38].

The impact of bleaching was not equivalent across MPAs and nMPAs. In 2015, the MPAs had less coral cover than the surrounding areas, while in 2021, the MPAs had a higher percentage of coral compared to nMPAs. This change in coral cover highlights a resilience effect of Moorea’s MPAs, an effect that can be effectively assessed using PAM, thus highlighting the importance of long-term monitoring. In the eastern sites, the resilience is clearly evident with PAM, whereas for the western sites, we can hypothesize that if the reduction of coral cover was too significant, more time is needed to observe a resilience effect. In addition, it is important to note that the massive bleaching event of 2019 caused mortality that is considered unprecedented (based on calculations at the Tiahura site since 1971) and has significant temporal inertia [96]. This means that in 2021, coral cover was still decreasing due to continued mortalities several years after the end of the abnormal temperatures, particularly affecting colonies that were partially dead in 2019 [96]. Concerning fish, declines in species richness and abundance of obligate corallivores were observed 3 years after mass coral mortality in Kiribati, while the abundance of facultative corallivores increased with disturbance [97].

### 4.2. MPAs versus nMPAs

In the high-frequency band (2–22 kHz), no differences in PSD_Fpeak_ were found between MPAs and nMPAs while a difference was observed for γFpeak. Transects showed that a difference in coral cover was present between MPAs and nMPAs in 2021 (21.7% versus 14.9%, respectively). We can hypothesize that this difference was not significant enough to be reflected acoustically when measuring only two parameters of the benthic invertebrates’ mass phenomena. When evaluating more distinct ecological states (e.g. pristine reef versus fished or bleached reef), PAM has proven effective at frequencies above 2 kHz with very short recording times (i.e. ≤20 min) at night to differentiate among coral reef ecological states [98]. In contrast, low-frequency biophony required recordings that were 10 times longer to discriminate among coral reefs’ ecological states [98].

In the low-frequency band (<2 kHz), used to assess fish mass phenomena, diurnal PSD_Fpeak_ was lower in MPAs and in sites with a higher algae cover. This may seem counterintuitive. The mass phenomena produced by fish may be less dependent on the health of the coral reef compared to the broadband transient sounds produced by snapping shrimps. It is known that Pomacentridae contribute significantly to the low-frequency mass phenomenon [65], and certain abundant vocal species (e.g. *Stegastes* spp.) primarily feed on filamentous algae [99], which can also be found on degraded reefs, but algae are not specifically associated with degraded reefs. There is also evidence of a lack of association between benthic cover and the densities of species such as *Stegastes nigricans* [100], a well-known soniferous Pomacentridae species. Finally, in Moorea, MPAs not only have a higher percentage of coral and fish species richness but also have a higher percentage of algae compared to nMPAs.

An overall less healthy reef than expected could also likely be due to violations of fishing regulations [37] that reduce the beneficial effect of protection measures. One of the observed differences between MPAs and nMPAs is a difference in γFpeak. γFpeak indicates the frequency at which this peak intensity occurs. It helps to determine how changes in the soundscape might affect species that rely on specific frequencies for communication or recruitment. This pattern may reflect differences in the species present responsible for distinct phenomena. Further investigation is needed to better understand this result. Indeed, although the ichthyofauna of Moorea is well documented through monitoring conducted by the Service National d’Observation CORAIL (http://observatoire.criobe.pf), more field studies on which species produce which sounds are still needed. In addition, to obtain more information about the link between fish sounds and the health of the reef, it is likely necessary to study the diversity of fish sounds and not only two features of their mass phenomena. Since PAM is known to enhance fish assemblage assessments during monitoring when used in conjunction with environmental data [101], it would be a valuable tool for monitoring coral reef MPAs. This has been demonstrated in temperate ecosystems, where PAM has been used in MPAs to detect species previously unknown in those areas [102,103]. Fish sound richness [58], as well as abundance and diversity [103], have also been shown to be higher in temperate MPAs compared to adjacent nMPAs, and these findings are reflective of taxonomic biodiversity assessments [58].

## 5. Conclusion

We hypothesized that the effects of bleaching would be less pronounced within MPAs due to their potential resilience in Polynesian scleractinian-dominated habitats. This was only observed in the high-frequency component of the biophony. High-frequency measurements of PSD and its associated peak frequency values demonstrated strong correlation with temporal changes in coral cover. Compared to fish sounds, benthic invertebrate sounds exhibit several advantages: (i) they are less masked by the geophony (which is crucial as the geophony level is known to increase due to a rise in storm frequency [104]) and the increasing anthropophony levels [105]; (ii) they more accurately reflect changes in coral cover; and (iii) they are easier to assess with automatic methods. We suggest including a measurement of nocturnal benthic invertebrate sounds in long-term coral reef monitoring studies due to its cost effectiveness and complementary nature with respect to classical monitoring methods. This will be particularly useful when traditional monitoring methods are difficult to implement or when enforcement is challenging.

## Data Availability

The data supporting the study are openly available on Zenodo [106–109]. In addition, more information can be found as part of the electronic supplementary material [110].
